# Circannual pattern of milk crude protein and urea nitrogen and their relationship in Holstein cows in Swiss grassland-based dairy systems over a 30-year period

**DOI:** 10.3168/jdsc.2026-1030

**Published:** 2026-06-11

**Authors:** Jedidiah Yi Lin Ong, Usman Arshad, Mutian Niu

**Affiliations:** Animal Nutrition, Institute of Agricultural Sciences, Department of Environmental Systems Science, ETH Zürich, Zürich 8092, Switzerland

## Abstract

The objectives of the study were to characterize the circannual variation in milk CP and MUN concentrations and to evaluate their relationship in Holstein cows managed under Swiss grassland-based dairy production systems. We hypothesized that milk CP and MUN exhibit distinct seasonal patterns, reflecting changes in forage quality and feeding practices, and that the strength of their association varies across seasons and production eras. A retrospective cohort study was conducted using historical milk recording data consisting of 2,313,348 cow-lactations from 897,854 unique Holstein cows across 22,830 Swiss dairy farms collected between 1994 and 2023. The dataset included primiparous (n = 817,638) and multiparous (n = 1,495,710) cow-lactations, with multiparous cows contributing a mean (±SD) lactation number of 3.04 ± 1.04. Monthly test-day records (January to December) within a 305-d lactation were used to assess temporal changes in milk CP (%) and MUN (mg/dL) and their relationship, resulting in approximately 18.3 million test-day observations. Mean (±SD) CP concentrations were 3.29% ±  0.34% for primiparous and 3.32% ± 0.36% for multiparous cows, and MUN concentrations were 10.8 ± 3.72 mg/dL and 10.5 ± 3.81 mg/dL, respectively. The study period was stratified into 2 eras: EraA (1994–2008) and EraB (2009–2023). Generalized linear mixed-effects models were fitted to assess temporal variation in milk CP and MUN and included fixed effects of test-day month (January to December), parity (primiparous vs. multiparous), era (EraA vs. EraB), their 2- and 3-way interactions, a natural cubic spline for DIM, and the interaction between parity and DIM, and random effects of farms and cow. To evaluate the relationship between milk CP and MUN, the model included fixed effects of linear and quadratic covariates of CP, parity, era, their 2- and 3-way interactions, a natural cubic spline for DIM and its interaction with parity, and random effects of farm nested within monthly test-day record and cow. A 3-way interaction between month, parity, and era was associated with milk CP and MUN concentrations, indicating that seasonal patterns differed between eras and that the magnitude and timing of these changes varied between primiparous and multiparous cows. Milk CP declined from winter to summer in both eras (EraA: primiparous 3.29–3.12, multiparous 3.34–3.18; EraB: primiparous 3.30–3.09, multiparous 3.36–3.12). In contrast, MUN concentrations increased from winter to late summer in both eras (EraA: primiparous 10.0–12.5, multiparous 9.82–12.2; EraB: primiparous 10.0–12.0, multiparous 9.71–11.8). A 3-way interaction between milk CP, parity, and era was observed for MUN, with MUN decreasing nonlinearly as CP increased from 2.40% to 4.20%, and steeper declines at lower CP. Across the milk CP range, multiparous cows generally exhibited slightly lower MUN than primiparous cows, with smaller parity differences in EraB. Overall, milk CP and MUN concentrations exhibited distinct circannual patterns across parity and production era, and MUN was associated with decline over time at similar CP levels, suggesting improved population-level N utilization, potentially associated with changes in nutrition, genetics, and forage management.

## Highlights

• Annual patterns of milk CP and MUN in Swiss Holstein cows were assessed with historic data.
• Production eras A (1994-2008) and B (2009-2023) were compared for long-term N dynamics.
• Multiparous cows showed consistently higher CP and lower MUN than primiparous cows.
• Milk CP decreased and MUN increased from winter to summer, less pronounced in EraB.
• Milk urea N declined over time at similar CP levels across parities.

## Graphical Abstract

**Figure:**
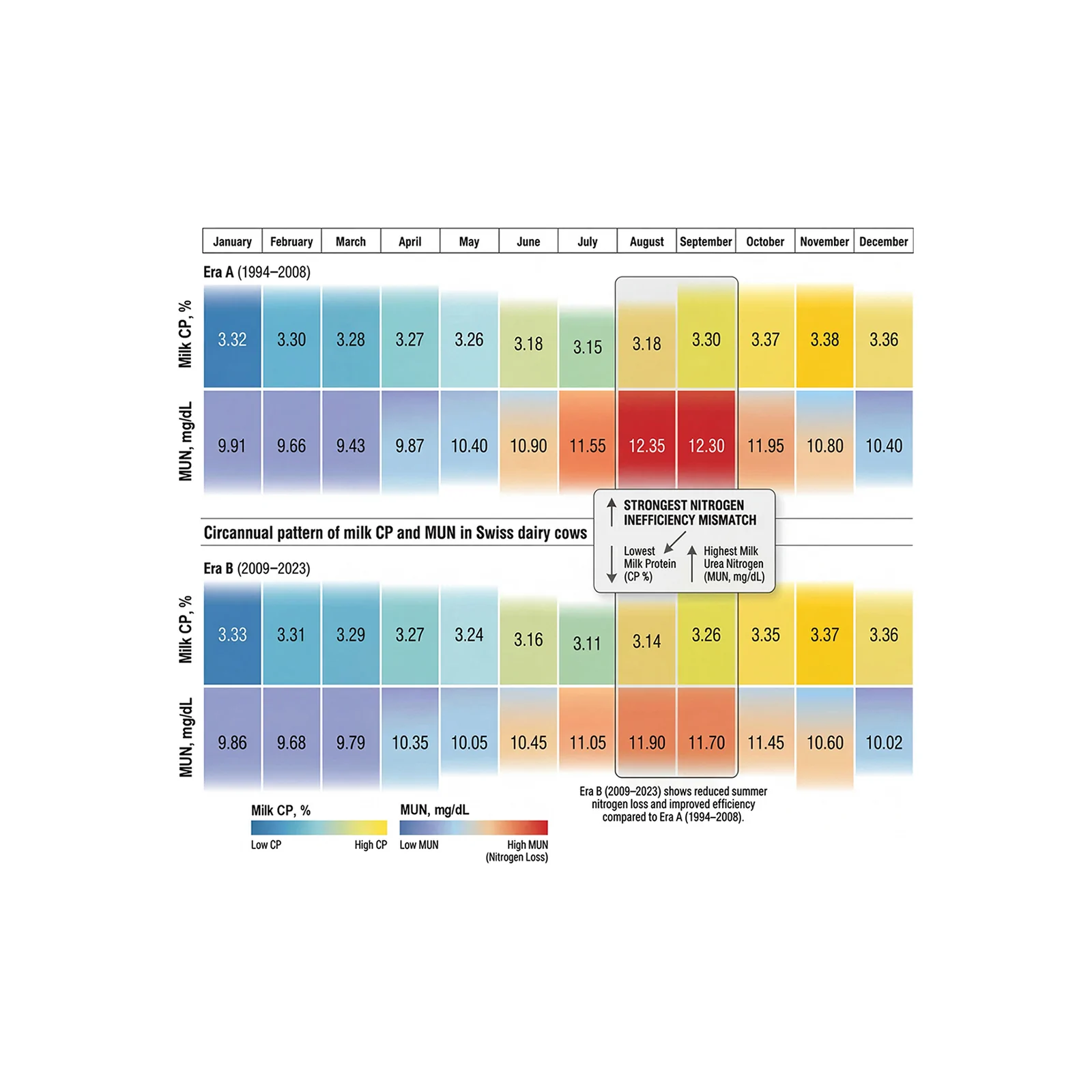
**Summary:** The objectives of this study were to characterize circannual variations in milk crude protein (CP) and milk urea nitrogen (MUN) and to evaluate their relationship in Holstein cows managed under grassland-based dairy systems using a large national dataset. Historical milk recording data of 2.3 million cow-lactations from 897,854 unique Holstein cows (including 817,638 primiparous and 1,495,710 multiparous cow-lactations) across 22,830 dairy farms in Switzerland, collected between 1994 and 2023, were analyzed. The study period was stratified into 2 eras: EraA (1994-2008) and EraB (2009-2023). Milk CP composition declined from winter to summer, whereas MUN increased, with smaller seasonal fluctuations in EraB. Multiparous cows consistently exhibited higher milk CP and lower MUN than primiparous cows across eras. The CP-MUN relationship was nonlinear, with steeper MUN declines at lower CP and reduced parity differences in EraB. These findings reveal MUN was associated with decline over time at similar CP levels, suggesting better N utilization across parities and eras.

Ruminants require dietary N to synthesize MP that supports essential physiological functions, including milk production, growth, and maintenance. However, N utilization of ruminants is variable and considered relatively low, with estimates typically ranging from 15% to 40% and averaging approximately 25% (Kohn et al., 2005; Calsamiglia et al., 2010). Nitrogen not captured in productive outputs is largely excreted in urine and feces, contributing to environmental concerns such as eutrophication and GHG emissions (Stark and Richards, 2008; Wattiaux et al., 2019). From a production standpoint, this inefficiency also has economic implications because dietary protein represents one of the most expensive components of dairy rations (de Freitas et al., 2019). Consequently, understanding factors associated with N partitioning in different dairy systems remains a priority for improving both environmental sustainability and economic performance.

Nitrogen use efficiency (**NUE**) is classically defined as the ratio of N output to N intake, but direct quantification of these components under commercial conditions is labor-intensive and rarely feasible at scale (Lavery and Ferris, 2021). As a result, indirect indicators have been widely adopted. Milk urea N reflects circulating urea derived from ruminal NH_3_ absorption and hepatic urea synthesis and is commonly interpreted as an indicator of N surplus relative to productive demands (Hof et al., 1997; Portnoy et al., 2021). Milk CP, in contrast, represents N captured in milk and is influenced by dietary supply, physiological state, and genetic potential. When evaluated jointly, CP and MUN may provide complementary information on N partitioning in lactating cows, without requiring direct measurements of N intake or excretion (Johnson and Young, 2003). However, it is importantly to note that these variables should be interpreted as indirect population-level indicators, rather than direct and precise estimates of NUE.

Previous studies have described associations between milk CP and MUN concentrations under experimental or short-term observational set-ups (Johnson and Young, 2003). Far fewer studies have examined how these traits, and their relationship, vary over extended time horizons. Long-term variation may reflect cumulative changes in genetic selection, feeding strategies, and climatic conditions (Keim and Anrique, 2011; Hill and Wall, 2015; Beatson et al., 2019), whereas short-term, within-year variation is largely driven by seasonal fluctuations in temperature, humidity, and forage quality (Heck et al., 2009; Oudah, 2009). These temporal dynamics may further differ by parity because primiparous and multiparous cows vary in production level and nutrient partitioning (Tavernier et al., 2023). Despite this, parity-specific assessments that jointly consider seasonal and long-term variation in milk CP and MUN remain limited.

These knowledge gaps are particularly relevant for grassland-based dairy production systems, which dominate milk production in many regions and are characterized by pronounced seasonal changes in forage composition and feeding practices (Keim and Anrique, 2011). Long-term national milk recording databases from such systems therefore provide a unique opportunity to examine population-level patterns in milk CP and MUN across months of the year and over multiple decades. Accordingly, the objectives of the present study were to characterize seasonal variation in milk CP and MUN concentrations and to evaluate their association in primiparous and multiparous Holstein cows managed under a grassland-based dairy production system, while also assessing whether these patterns differed between production eras. We hypothesized that milk CP and MUN exhibit distinct circannual patterns and that the strength of their association varies by parity and production era.

This study was conducted at the Institute of Agricultural Sciences, Department of System Sciences, ETH Zürich, Switzerland. No experimental procedures involving live animals were performed; therefore, in accordance with institutional and national guidelines, ethical approval and animal care licenses were not required. Historical milk recording data from the Swiss Holstein Breeding Association were analyzed in a retrospective cohort design, consisting of 2,313,348 cow-lactations (primiparous: n = 817,638; multiparous: n = 1,495,710) from 897,854 unique Holstein cows across 22,830 Swiss dairy farms, collected between 1994 and 2023. Multiparous cows contributed to the dataset with a mean (±SD) lactation number of 3.04 ± 1.04, ranging from 2 to 5. The herd size of Holstein cows averaged (±SD) 12.3 ± 14.3 per farm-year (median = 8; range: 1–420). Although Swiss farms may include multiple breeds (e.g., Brown Swiss, Original Braunvieh, Swiss Fleckvieh, Jersey, and Simmental), the present descriptive statistics of herd dynamics represent only Holstein cows. Although participation in official breeding programs was voluntary, the extensive geographic coverage and large sample size provided a broad representation of Swiss dairy production systems. To account for temporal changes in dairy production systems, the study period (1994–2023) was partitioned into 2 eras: **EraA** (1994–2008) and **EraB** (2009–2023). The EraA represents an earlier production phase preceding major modernization, whereas EraB reflects more recent production conditions characterized by improvements in herd management, nutritional strategies, and genetic selection. The definition of these eras was informed by exploratory analyses of temporal structure. Specifically, mixed-effects models treating year as a continuous variable indicated a gradual but consistent downward shift in the CP–MUN relationship over time (Supplemental Figure S1, see Notes), and unsupervised clustering of annual CP–MUN profiles (based on means, variability, and seasonal contrasts) identified 2 distinct groups of eras corresponding broadly to earlier and later periods, with a transition occurring in the mid-to-late 2000s (Supplemental Figure S2, see Notes). Based on these complementary analyses, a cutoff around 2008 and 2009 was selected to represent the transition between historical and more recent production conditions. The era-based specification was retained in the main models to provide a parsimonious and biologically interpretable representation of temporal effects, while avoiding overparameterization associated with highly flexible continuous-time interactions. Data collection was primarily motivated by herd improvement and performance evaluation, resulting in a sampling structure that was inherently sparse and less uniform than protocols typical of experimental studies. Monthly test-day samples (January to December) were collected from participating farms following standardized guidelines. For each cow-lactation, a single observation per month was used to assess temporal changes in milk CP and MUN. In cases where more than 1 test-day sample was available within the same month for a given cow-lactation, the values were averaged, so that each cow-lactation contributed only 1 CP and MUN measurement per month in a 305-d lactation. Overall, this approach resulted in approximately 18.3 million test-day records included in the analysis. A multistage data-cleaning procedure was applied before statistical analysis. Outliers in milk CP (%), and urea (mg/dL) were removed using an interquartile range (**IQR**) filter applied by year. Samples were flagged as outliers if any of the 2 variables fell outside the range Q1 − 1.5 × IQR to Q3 + 1.5 × IQR, where Q1 and Q3 are the first and third quartiles, respectively. Only samples with simultaneous milk CP and urea measurements across lactations were retained. Data were excluded if they originated from research farms, educational institutions, or breeding organizations; if samples had DIM greater than 305 d; or if farms contributed fewer than 1,000 samples. Records involving stillbirths, abortions, or other unsuccessful calving events were removed, and only cows with at least 6 unique test-day records per cow-lactation were retained to ensure adequate coverage of the standard 305-d lactation period. Milk urea values were converted to MUN as follows:

MUN (mg/dL) = milk urea (mg/dL) × 0.466.

All statistical analyses were performed in R (R version 4.1.2; R Core Team, 2021) using generalized linear mixed-effects models implemented with the *lmer* procedure (Bates et al., 2015). Associations between concentrations of milk CP or MUN and the month of test-day sampling were analyzed to assess temporal changes using the following mixed model:

Y*_ijklm_* = u + Month*_i_* + Parity*_j_* + Era*_k_* + (Month*_i_* × Parity*_j_*) + (Month*_i_* × Era*_k_*) + (Parity*_j_* × Era*_k_*) + (Month*_i_* × Parity*_j_* × Era*_k_*) + f_DIM_ (DIM*_i_*) + f_DIM_(DIM*_i_*) × Parity*_j_* + u*_l_* + v*_m_* + ε*_ijklm_*,

where Y*_ijklm_* = response of interest (CP or MUN) for month *i*, parity group *j*, era *k*, farm *l*, and cow *m*; u = intercept; u*_l_* = farm; v*_m_* = cow; and ε*_ijklm_* = residuals. In order to evaluate the relationship between MUN and CP, the following statistical model was fitted:

$$[\begin{array}{l} Y_{ijklm}=u+CP_{i}+{CP}_{i}^{2}+Parity_{j}+Era_{k}+\left(CP_{i}\times Parity_{j}\right)+\\ \left({CP}_{i}^{2}\times Parity_{j}\right)+\left(CP_{i}\times Era_{k}\right)+\left({CP}_{i}^{2}\times Era_{k}\right)+\\ \left(Parity_{j}\times Era_{k}\right)+\left(CP_{i}\times Parity_{j}\times Era_{k}\right)+\left({CP}_{i}^{2}\times Parity_{j}\times Era_{k}\right)\\ +f_{DIM}\left(DIM_{ijklm}\right)+f_{DIM}\left(DIM_{ijklm}\right)\times Parity_{j}+u_{l}+v_{m}+\varepsilon_{ijklm}, \end{array}$$

where Y*_ijklm_* = response of interest (MUN) for observation *i*, parity group *j*, era *k*, farm-month *l*, and cow *m*; u = intercept; u*_l_* = farm-month, v*_m_* = cow, and ε*_ijklm_* = residuals. In both models, DIM was modeled as a natural cubic spline (df = 5), and its interaction with parity was included to allow for parity-specific nonlinear lactation curves.

Variable selection was conducted using backward elimination, removing the highest-order terms first when *P* > 0.10, whereas all other covariates were retained in the final models. Normality of residuals and homogeneity of variance were assessed for each continuous dependent variable after fitting the statistical model. An iterative outlier removal procedure was implemented. In each iteration, studentized residuals were calculated, and records exceeding ±3 were removed from the dataset. This procedure was repeated until no residuals exceeded ±3 or a maximum of 10 iterations was reached. The Kenward–Roger method was used to obtain the approximate denominator df for the *F*-tests of all statistical models. Variance inflation factor was also calculated to assess multicollinearity among fixed effects after fitting the statistical models. In addition to analyses on the actual CP scale (%), we computed a within-lactation centered CP variable for sensitivity and interpretability as

$$CP\_C_{ij}=CP_{ij\;}-{\bar{CP}}_{j},$$

where ${\bar{CP}}_{j}$ denotes the mean CP for cow-lactation *j*. Because centering did not change the interpretation of the results, all statistical analysis were conducted and presented on the actual CP scale for ease of interpretation. Figures were created as scatter plots with the response of interest on the Y-axis and explanatory variables (month or CP) on the x-axis, using the predicted responses for each cow-lactation contributing data to the statistical analysis of the dependent variable. Significance was declared when *P* ≤ 0.05 and a tendency to differ at 0.05 < *P* ≤ 0.10.

Descriptive statistics for cow-lactations, monthly test-day records, DIM, and milk CP and MUN concentrations by parity and era are summarized in Table 1. A 3-way interaction between month, parity, and era was associated (*P* < 0.01) with concentrations of milk CP, indicating that seasonal patterns differed between EraA and EraB and that the magnitude and timing of these seasonal changes varied between primiparous and multiparous cows within each era (Figure 1A). In EraA, concentrations of milk CP declined from winter to summer months, with primiparous cows decreasing from 3.29% in January to 3.12% in July and multiparous cows from 3.34% to 3.18%, before rising steadily to 3.32% and 3.39%, respectively, by December. In EraB, primiparous cows showed a slightly smaller summer decline (3.30% to 3.09%) and a rise to 3.32% in December, whereas multiparous cows exhibited a more pronounced summer reduction (3.36% to 3.12%) with recovery to 3.39% by year-end. Multiparous cows consistently maintained higher milk CP than primiparous cows in both eras, and the magnitude and timing of seasonal changes differed between eras and parity groups (Figure 1A). A 3-way interaction between month, parity, and era was also observed (*P* < 0.01) with concentrations of MUN, indicating that seasonal patterns differed between EraA and EraB and that the magnitude and timing of these seasonal changes varied across parities within each era (Figure 1B). In EraA, MUN concentrations increased from winter to late summer months, with primiparous cows rising from 10.0 mg/dL in January to 12.5 mg/dL in August before declining to 10.6 mg/dL by December, and multiparous cows increasing from 9.82 mg/dL to 12.2 mg/dL over the same period, followed by a decrease to 10.2 mg/dL by year-end. In contrast, in EraB, primiparous cows showed a more moderate summer increase (10.0 to 12.0 mg/dL) with a decline to 10.2 mg/dL in December, whereas multiparous cows experienced a smaller rise (9.71 to 11.8 mg/dL) and a recovery to 9.84 mg/dL by December (Figure 1B). A 3-way interaction between linear covariate of CP, parity, and era was observed (*P* < 0.01) for MUN, indicating that the CP and MUN relationship differed between EraA and EraB and between primiparous and multiparous cows (Figure 1C). In EraA, MUN decreased as milk CP increased from 2.40% to 4.20%, with primiparous cows dropping from 11.7 mg/dL of MUN at 2.50% CP to 11.1 mg/dL at 3.50% CP and multiparous cows from 11.5 to 10.9 mg/dL of MUN. In EraB, primiparous cows showed a slightly smaller decrease in MUN (11.3 to 10.9 mg/dL), and multiparous cows had a more modest reduction (11.0 to 10.7 mg/dL). Slopes of MUN were steeper at lower CP and flattened at higher CP, reflecting the quadratic response to CP. Across the full CP range (2.40% to 4.20%), multiparous cows generally associated with slightly lower MUN than primiparous cows in EraA, and differences were smaller in EraB.

**Table 1:** Summary statistics of cow-lactations, monthly test-day records, DIM, and concentrations of CP and MUN according to different parity and era in Swiss grassland-based Holstein dairy cows

Item	Primiparous				Multiparous			
	Mean ± SD	Median	Minimum	Maximum	Mean ± SD	Median	Minimum	Maximum
EraA (1994–2008)								
Cow-lactations, n	333,161	—	—	—	542,717	—	—	—
Monthly test-day, n	2,687,116	—	—	—	4,251,783	—	—	—
Records per cow-lactation, n	8.07 ± 0.93	8.00	6.00	15.0	7.83 ± 1.00	8.00	6.00	15.0
DIM	148 ± 84	147	1.00	305	147 ± 83	146	1.00	305
CP, %	3.27 ± 0.31	3.25	2.40	4.20	3.30 ± 0.34	3.28	2.40	4.20
MUN, mg/dL	11.3 ± 3.6	11.2	3.26	21.0	11.2 ± 3.6	11.2	3.26	21.0
EraB (2009–2023)								
Cow-lactations, n	484,477	—	—	—	952,993	—	—	—
Monthly test-day, n	3,884,497	—	—	—	7,457,803	—	—	—
Records per cow-lactation, n	8.02 ± 0.99	8.00	6.00	14.0	7.83 ± 1.03	8.00	6.00	15.0
DIM	151 ± 84	151	1.00	305	151 ± 83	151	1.00	305
CP, %	3.29 ± 0.34	3.28	2.40	4.20	3.32 ± 0.36	3.31	2.40	4.20
MUN, mg/dL	10.8 ± 3.7	10.7	3.26	21.0	10.5 ± 3.8	10.3	3.26	21.0

**Figure 1:**
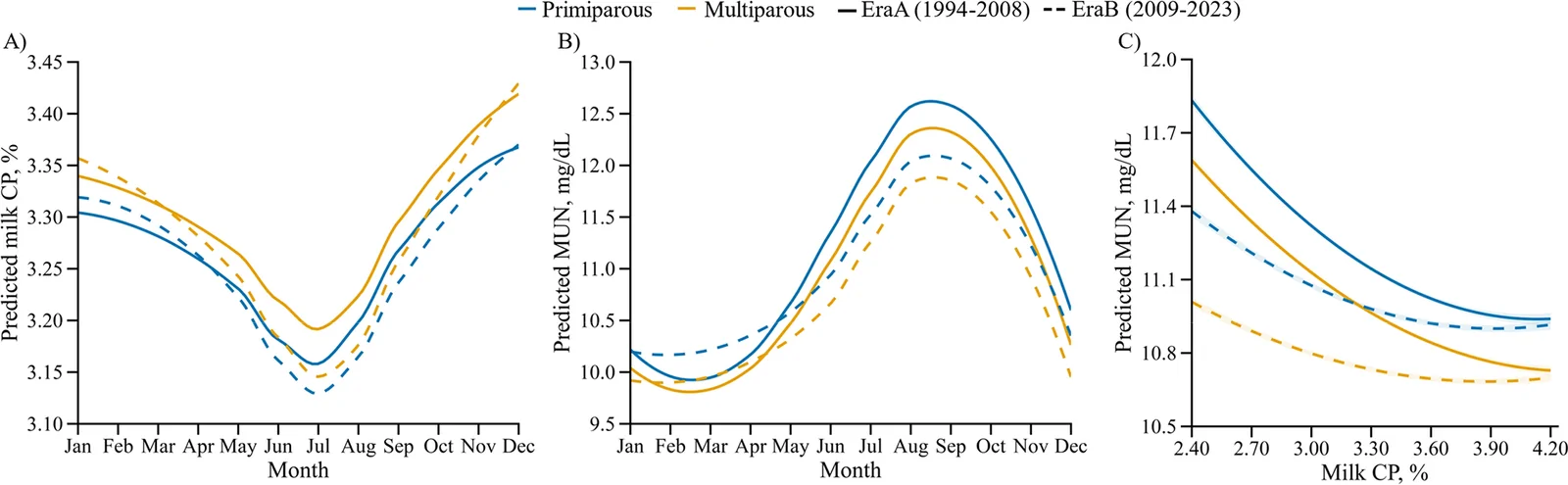
Panels A and B present predicted milk CP (A) and MUN (B) according to month in primiparous and multiparous Holstein cows from era A (EraA; 1994–2008) and B (EraB; 2009–2023), respectively. The maximal SEM values observed for MUN and CP across both parity and era were 0.01 and 0.001, respectively. Panel C indicates the association between MUN and CP in primiparous and multiparous Holstein cows from EraA and EraB in grassland-based production system; the shaded region indicates 95% CI. Panel A and B: associations of month (*P* < 0.01), parity (*P* < 0.01), era (*P* < 0.01), and 3-way interaction between month, era and parity (*P* < 0.01). Panel C: associations of linear (*P* < 0.01) and quadratic (*P* < 0.01) covariate of CP, parity (*P* < 0.01), era (*P* < 0.01), and 3-way interaction between linear covariate of CP, era, and parity (*P* < 0.01).

Seasonal variation in milk CP and MUN generally occurred in opposite directions, with milk CP increasing as MUN declined from August to September, and the reverse pattern observed from January through July. This pattern is consistent with the inverse association between milk CP and MUN identified in the association models, although seasonal trajectories were not perfectly synchronized. Notably, milk CP reached its annual minimum in July, whereas MUN peaked slightly later in August. Several studies have reported increased MUN (Roths et al., 2023; Tshuma et al., 2023) or decreased milk CP (Bertocchi et al., 2014; Chen et al., 2024) during the summer, but few have examined these responses together. Consequently, the partial decoupling of seasonal changes in milk CP and MUN remains difficult to mechanistically resolve. One plausible explanation is that milk CP and MUN respond to environmental stressors on different timescales. Milk CP may respond relatively rapidly to heat stress through reductions in feed intake and AA availability and altered endocrine regulation, including lower circulating insulin concentrations (Rhoads et al., 2009; Chen et al., 2024). In contrast, MUN may reflect more indirect and integrated processes, including heat stress-induced alterations in rumen fermentation patterns and microbial N utilization (Zhao et al., 2019), as well as postabsorptive N metabolism, which together influence the balance between urea production and recycling. Because these processes may operate on different timescales, changes in MUN could lag behind changes in milk CP, contributing to the observed temporal offset.

A downward shift in both CP and MUN concentrations in EraB relative to EraA was evident from May through November in both parity groups, whereas from January through early spring, differences between eras were less consistent. The reductions in milk CP during summer and autumn in EraB coincided with a period characterized by higher ambient temperatures, as mean conditions from May to November were approximately 0.80°C warmer compared with EraA (Begert and Frei, 2018). These findings are consistent with previous reports that heat stress can reduce milk protein concentration through mechanisms not solely related to N supply, including greater diversion of AA toward maintenance functions and reduced mammary protein synthesis efficiency (Guo et al., 2018). As a result, even if systemic changes over time were associated with lower MUN concentrations, this did not necessarily translate into higher milk CP during periods of elevated thermal stress. Parity-specific differences were also evident across the course of a year and over years. Multiparous cows consistently exhibited higher milk CP and lower MUN concentrations than primiparous cows, a pattern that was also apparent in the CP–MUN association models (Fatehi et al., 2012). The persistence of the parity gap across months indicates that seasonal fluctuations in forage quality and environmental conditions might not differentially affect primiparous and multiparous cows. Rather, these differences might be consistent with intrinsic physiological distinctions between parity classes. Primiparous cows are still undergoing growth and therefore partition nutrients, particularly protein, between mammary tissue for milk synthesis and body tissue for ongoing development. In contrast, multiparous cows, having largely completed growth, can direct a greater proportion of absorbed protein toward milk production.

Across the full dataset, the association between milk CP and MUN was shown to be nonlinear, with MUN declining as CP increased before reaching a plateau at higher CP concentrations. This population-level pattern may be biologically consistent with established principles of ruminant N metabolism, whereby greater capture of N in milk CP is associated with lower circulating urea concentrations up to a physiological limit (Broderick and Clayton, 1997; Nousiainen et al., 2004). At lower milk CP levels, steeper declines in MUN may reflect more synchronized rumen fermentation and microbial N utilization, whereas the plateau observed at higher CP levels likely reflects limits to AA utilization, beyond which excess N is deaminated and converted to urea. Importantly, this interpretation reflects associations observed at the population level and does not imply direct measurement of N intake or excretion. The consistent downward displacement of MUN across eras at comparable milk CP concentrations suggests a potential long-term shift in N partitioning patterns in Swiss dairy herds. This temporal trend is consistent with reported changes in dairy production systems, including the adoption of more precise feeding strategies, improved balancing of protein and energy supply, and advances in forage quality and conservation practices (Peyraud and Delagarde, 2013; Gislon et al., 2020). Genetic selection for milk solids and feed efficiency traits may have further contributed to these patterns. In this study, direct information on dietary composition, nutrient intake, or N excretion was not available, therefore, these interpretations should be viewed as consistent with, rather than demonstrating, potential improvements in NUE. Parity differences in MUN were also evident across eras, with multiparous cows exhibiting lower MUN concentrations than primiparous cows, consistent with a previous observational study (Fatehi et al., 2012).

Several limitations should be acknowledged. This retrospective analysis included more than 19 million test-day records from approximately 2.30 million cow-lactations across 22,830 farms over a 30-yr period, during which substantial changes occurred in management, genetics, and analytical methodologies. Consequently, the observed temporal patterns likely reflect the combined influence of multiple evolving factors, including feeding practices, forage composition, genetic merit, and milk analysis procedures. As an observational study, causal relationships cannot be inferred, and residual confounding may remain despite extensive model adjustment. Moreover, the lack of detailed information on diet composition, feed intake, genetics, and N excretion limits the ability to identify the specific biological mechanisms underlying changes in MUN and milk CP over time.

Despite these limitations, the consistency of associations across seasons, parity groups, and production eras supports the biological plausibility of long-term shifts in N partitioning patterns in grassland-based dairy systems. The observed reduction in MUN at comparable milk CP concentrations suggests that modern production systems may differ systematically from earlier decades in how N is utilized and expressed in milk traits. From a sustainability perspective, these population-level trends are consistent with the possibility of reduced N surplus, although direct quantification of environmental N losses was beyond the scope of this study. Moreover, the circannual decoupling of milk CP and MUN highlights that improvements in N partitioning patterns may not always coincide with higher milk protein concentrations, particularly under conditions of heat stress. Future studies integrating climatic, dietary, genetic, and intake data will be essential to disentangle these interacting influences and to better understand how environmental stressors shape NUE in dairy cows.
